# Host-Induced Silencing of Two Pharyngeal Gland Genes Conferred Transcriptional Alteration of Cell Wall-Modifying Enzymes of *Meloidogyne incognita vis-à-vis* Perturbed Nematode Infectivity in Eggplant

**DOI:** 10.3389/fpls.2017.00473

**Published:** 2017-03-30

**Authors:** Tagginahalli N. Shivakumara, Sonam Chaudhary, Divya Kamaraju, Tushar K. Dutta, Pradeep K. Papolu, Prakash Banakar, Rohini Sreevathsa, Bhupinder Singh, K. M. Manjaiah, Uma Rao

**Affiliations:** ^1^Division of Nematology, Indian Council of Agricultural Research – Indian Agricultural Research InstituteNew Delhi, India; ^2^Indian Council of Agricultural Research – National Research Centre on Plant BiotechnologyNew Delhi, India; ^3^Nuclear Research Laboratory, Indian Council of Agricultural Research – Indian Agricultural Research InstituteNew Delhi, India; ^4^Division of Soil Science and Agricultural Chemistry, Indian Council of Agricultural Research – Indian Agricultural Research InstituteNew Delhi, India

**Keywords:** HIGS, *msp-18*, *msp-20*, ^14^C isotope, RNAi, CWME, qRT-PCR

## Abstract

The complex parasitic strategy of *Meloidogyne incognita* appears to involve simultaneous expression of its pharyngeal gland-specific effector genes in order to colonize the host plants. Research reports related to effector crosstalk in phytonematodes for successful parasitism of the host tissue is yet underexplored. In view of this, we have used *in planta* effector screening approach to understand the possible interaction of pioneer genes (*msp-18* and *msp-20*, putatively involved in late and early stage of *M. incognita* parasitism, respectively) with other unrelated effectors such as cell-wall modifying enzymes (CWMEs) in *M. incognita*. Host-induced gene silencing (HIGS) strategy was used to generate the transgenic eggplants expressing *msp-18* and *msp-20*, independently. Putative transformants were characterized via qRT-PCR and Southern hybridization assay. SiRNAs specific to *msp-18* and *msp*-*20* were also detected in the transformants via Northern hybridization assay. Transgenic expression of the RNAi constructs of *msp-18* and *msp-20* genes resulted in 43.64–69.68% and 41.74–67.30% reduction in *M. incognita* multiplication encompassing 6 and 10 events, respectively. Additionally, transcriptional oscillation of CWMEs documented in the penetrating and developing nematodes suggested the possible interaction among CWMEs and pioneer genes. The rapid assimilation of plant-derived carbon by invading nematodes was also demonstrated using ^14^C isotope probing approach. Our data suggests that HIGS of *msp-18* and *msp-20*, improves nematode resistance in eggplant by affecting the steady-state transcription level of CWME genes in invading nematodes, and safeguard the plant against nematode invasion at very early stage because nematodes may become the recipient of bioactive RNA species during the process of penetration into the plant root.

## Introduction

Plant-parasitic root-knot nematode (RKN: *Meloidogyne* spp.) causes substantial yield losses in various agri- and horticultural crops (Jones et al., 2013; Saucet et al., 2016). The infective second stage juvenile (J2) of RKN penetrates the host root apex and migrates intercellularly until it reaches the differentiating vascular tissue. During penetration, RKN secretes a repertoire of effector molecules of pharyngeal gland origin in the host-nematode interface via the stylet orifice. These effectors are thought to play essential roles in plant–nematode interaction starting from the host recognition process to softening of plant cell walls in order to facilitate the migration of nematode, selecting a suitable feeding site in vascular cylinder and establishing feeding cells which serves as the potential sink for nutrients that are necessary for nematode development and reproduction (Gheysen and Mitchum, 2011; Hewezi and Baum, 2013). During the last two decades, plethora of effector genes have been identified using various strategies such as cDNA-AFLP, cDNA library screening, bioinformatic mining, transcriptome and genome sequencing, and proteomic studies (Mitchum et al., 2013; Niu et al., 2016). However, only a small percentage of these parasitism genes have been functionally elucidated in detail. Based on their putative function, abstractly, phytonematode effectors can be categorized into the following divisions: (a) chemotactic factors (HYP and MAP-1 proteins are secreted from amphids, Rosso et al., 2011; Eves-van den Akker et al., 2014) possibly involved in host location and finding suitable feeding sites; (b) cell-wall modifying enzymes (CWMEs) that aid in nematode invasion and migration into and within hosts; (c) host reprogramming modulators associated in the initiation and maintenance of feeding cells; (d) putative host metabolism regulators to provide nutrients to nematodes; and (e) immunosuppressive components to protect nematodes and feeding cells against host innate defense responses (Mantelin et al., 2017).

Currently, we have only a rudimentary understanding on the expression profile of nematode effectors at the host-nematode interface. Determining the specific triggers for the activation and repression of different effectors throughout the parasitic process and their site of function in host cells is central to completely unravel the mysteries of nematode parasitism in plants. It is assumed that the concurrent expression of several effectors is required in order to successfully parasitize the host plants by nematodes (Mitchum et al., 2013), although to support this notion enough experimental proofs are not yet available. Recently, using *in vitro* and *in vivo* reverse-genetics approach we have demonstrated (Shivakumara et al., 2016) the existence of crosstalk among various *Meloidogyne incognita* effectors the origin of which encompass both the subventral (CWMEs – xylanases: *Mi-xyl-1*, *Mi-xyl-3*, β-1,4-endoglucanase: *Mi-eng-1*, pectate lyase: *Mi-pel*, polygalacturonase: *Mi-pg-1*, Haegeman et al., 2012; pioneer gene – *msp-20*, Huang et al., 2003) and dorsal esophageal glands (pioneer genes: *msp-16*, *msp-18*, *msp-24*, *msp-33*; Huang et al., 2003). It was shown that the induced suppression of one effector gene (subventral gland-specific) leads to the transcriptional alteration of other unrelated effectors (dorsal gland-specific) and vice versa in pre-parasitic J2s of *M. incognita*. In addition, knockdown of pioneer genes (supposedly play minimal role in nematode migration) led to the reduced penetration ability of *M. incognita* in tomato (Shivakumara et al., 2016). These findings suggested that there is a potential link between the expression of subventral gland-specific CWMEs and dorsal gland-specific pioneer genes during early stage of nematode infection process. Notably, nematode subventral glands play major role during the penetration and migration of host tissue (early infection process) whereas dorsal glands putatively function during the initiation and maintenance of the feeding site (late infection process; Gheysen and Mitchum, 2011).

It was suggested that the *in planta* effector screening approach is one of the potential means to unravel the intricacies of effector–effector interaction at the host-nematode interface (Mitchum et al., 2013). During the last decade, host-induced gene silencing (HIGS) has emerged as a useful tool for screening of various nematode-derived genes *in planta* (Dutta et al., 2015a,b). Using HIGS approach, multitude of effector genes have been functionally validated in detail in various host plants. Among the pioneer genes, *msp-16*/peptide16D10 was shown to be crucial for RKN infectivity and pathogenicity in *Arabidopsis* (Huang et al., 2006), grapevines (Yang et al., 2013), and potato (Dinh et al., 2014). HIGS of *msp-9* gene had impaired the infection ability of *M. incognita* in *Arabidopsis* (Xue et al., 2013). Similarly, HIGS of *msp-12* (Xie et al., 2016) and *msp-40* (Niu et al., 2016) attenuated the parasitic ability and fecundity of *M. incognita* in *Nicotiana benthamiana* and *Arabidopsis*. Nevertheless, none of these studies have analyzed the effect of *in planta* effector suppression on expression of other effectors (especially those which play key role during early stages of nematode parasitism such as CWMEs).

In the present study, HIGS approach was employed to express two important pioneer genes, i.e., *msp-18* and *msp-20*, specific to dorsal and subventral esophageal glands of *M. incognita*, respectively, in eggplant. Both the first and second generation transgenic lines for each gene exhibited resistance to *M. incognita* in terms of significant reduction in nematode multiplication. Intriguingly, a substantial reduction in nematode penetration was also documented in those transgenic lines for both *msp-18* (putatively play minimal role in nematode penetration process) and *msp-20* genes. The transgenic delivery of dsRNA (double-stranded RNA)/siRNA (small interfering RNA) of both *msp-18* and *msp-20* had caused transcriptional alteration of unrelated effectors such as CWMEs in feeding juveniles and females of *M. incognita*, as revealed by the quantitative reverse-transcription PCR (qRT-PCR) assay. Assuming that the cell sap containing bioactive RNA species (in the form of dsRNA/siRNA) may translocate from plant to nematode through amphidial aperture or stylet lumen during the nematode penetration process (viz., well before the initiation of a feeding cell), in the current study, we have labeled the wild-type eggplant with ^14^CO_2_ (Kölling et al., 2013; Singh et al., 2014) and challenge inoculated the labeled plants with *M. incognita* J2. Qualitative analysis of carbon allocation from source (shoot) to sink (root) tissues was done via autoradiogram generation and quantitative measurement of incorporated radioactivity in nematode juvenile was carried out via liquid scintillation counting. This proof-of-concept experiment indicated the traces of ^14^C isotope in the nematode J2 latching on to root tip prior penetration, which validates the hypothesis that nematodes become the recipient of plant-derived molecules during their exploratory probing phase on the plant surface.

## Materials and Methods

### Culturing of Nematodes

A pure culture of *M. incognita* race 1 was maintained on eggplant (*Solanum melongena* L. cv. Pusa Purple Long) in a glasshouse. Egg masses were extracted from the roots of 2-months-old plant using sterilized forceps and were hatched in a Petri dish containing sterile water via modified Baermann assembly (Whitehead and Hemming, 1965). Freshly hatched J2s were used for all the experiments. Other life stages were carefully dissected out of the infected tissue at different intervals for qRT-PCR assay.

### Cloning of Candidate Genes from *M. incognita*

Total RNA (400 ng) was isolated from the pre-parasitic J2 of *M. incognita*, their quality and quantity were assessed and converted to cDNA as described by Shivakumara et al. (2016). Fragments of five CWMEs (*Mi-xyl-1*: AF224342, *Mi-xyl-3*: EU475876, *Mi-pg-1*: AY098646, *Mi-eng-1*: AF100549, and *Mi-pel*: AF527788) and two pioneer genes (*msp-18*: AY134437 and *msp-20*: AY134439) were PCR amplified, cloned into pGEM-T vector and identity of the insert was confirmed via sequencing as described in Shivakumara et al. (2016). Primer details are documented in Shivakumara et al. (2016).

### Comparative Expression of CWME and Pioneer Genes in Different Life Stages of *M. incognita*

Differential expression of each of the *msp-18*, *msp-20*, *Mi-xyl-1*, *Mi-xyl-3*, *Mi-pg-1*, *Mi-eng-1*, and *Mi-pel* transcripts were analyzed by qRT-PCR at six different developmental stages (eggs, pre-parasitic J2, post-parasitic J2, J3/J4, young female and adult female) of *M. incognita*. RNA was extracted from different stages of *M. incognita* and reverse transcribed to cDNA as described above. All qRT-PCR primers (Shivakumara et al., 2016) were designed using IDT OligoAnalyzer 3.1 software^1^ and optimized for working concentration and annealing temperature prior to use with a Realplex^2^ thermal cycler instrument (Eppendorf). The 10 μl reaction mixture contained 1.5 ng of cDNA, 750 nM of each primer and 5 μl of SYBR Green PCR master-mix (Eurogentec). The amplification reaction conditions and melt curve program were set as described by Shivakumara et al. (2016). Cycle threshold (Ct) values were exported from Realplex^2^ software (Eppendorf). Gene expression was normalized using *18S rRNA* (Genbank accession number HE667742) as reference and fold change in expression was calculated according to the augmented comparative Ct method (Livak and Schmittgen, 2001) and log_2_-transformed. At least three biological and three technical replicates were used for each of the samples.

### Development of *msp-18* and *msp-20* RNAi Constructs for HIGS

The pK7GWIWG2(I) binary vector (RNAi Gateway ready) was obtained from VIB Department of Plant Systems Biology, Ghent University, Belgium^2^. Partial sequences of *msp-18* (456 bp) and *msp-20* (598 bp) flanked by attB1 and attB2 sites were PCR amplified from respective pGEMT clones and sub-cloned into the entry vector (pDONR 221) followed by transfer to pK7GWIWG2(I) in sense and antisense orientation (Supplementary Figure S1). The recombinant clones were transformed into *Agrobacterium tumefaciens* (LBA4404) competent cells using electroporation technique, positive clones were maintained in selection medium following standard techniques. Detailed procedure is described in Papolu et al. (2013) and Dutta et al. (2015b). Primer details are given in Supplementary Table S1.

### Co-transformation and Selection of Explants

Eggplant (cv. Pusa Purple Long) seeds were surface sterilized and germinated on MS agar medium (pH 5.8). Leaf explants of 1 cm^2^ cut from the fortnight-old eggplant seedling were used for *Agrobacterium*-mediated transformation. Pre-cultivated explants were infected with *A. tumefaciens* (LBA4404) cells harboring the RNAi constructs of *msp-18* and *msp-20*. Agroinfected leaves were plated on co-cultivation media followed by transfer to the selection medium containing kanamycin. Shoots that emerged from the explants were excised and sub-cultured in fresh selection medium. Elongated shoots were cultured in rooting media supplemented with 0.1 mg L^-1^NAA (Supplementary Figure S2). Plants with well-established roots were hardened and transferred to the transgenic glasshouse facility at ICAR-IARI, New Delhi till the production of T_0_ seeds. Plants transformed with *A. tumefaciens* harboring empty construct, and thus underwent no kanamycin selection, served as the control.

### Detection of Transgene Using PCR and Southern Hybridization

Genomic DNA was isolated from the fresh leaves of all the primary events (T_0_) using a Nucleospin plant II DNA extraction kit (Macherey-Nagel) following the manufacturer’s instructions. All the putative transformants were initially confirmed using standard PCR reaction with different sets of primers which are enlisted in Supplementary Table S1. PCR products were resolved on 1.2% agarose gel. In order to analyse the integration and copy number of *msp-18* and *msp-20* transgenes in PCR-confirmed T_0_ plants, Southern hybridization was carried out. Genomic DNA (15 μg) of each event was digested with *Sac*I enzyme (cuts once in the T-DNA), electrophoresed on 0.8% agarose gel and transblotted to a nitrocellulose membrane (Bio-Rad). For probing, a 456 bp fragment of the *msp-18* gene and a 598 bp of the *msp-20* gene were used independently. Probe labeling, hybridization and blot development were performed as described in Papolu et al. (2013) and Dutta et al. (2015b).

### Detection of Transgene Expression in Eggplant Using qRT-PCR and Northern Hybridization

PCR-positive T_0_ plants were allowed to self-pollinate and T_1_ seeds were obtained. Surface-sterilized T_1_ seeds were germinated in MS medium containing kanamycin (100 mg L^-1^) followed by transfer to pots filled with autoclaved soil. To analyse the transcript abundance of *msp-18* and *msp-20* genes in T_1_ plants, qRT-PCR experiment was carried out. Total RNA was isolated from the fresh leaves of PCR-positive T_1_ events using a Nucleospin plant II RNA extraction kit (Macherey-Nagel) and reverse transcribed to cDNA using a cDNA synthesis kit (Superscript VILO, Invitrogen) following the manufacturer’s instructions. qRT-PCR experiment was conducted with three independent biological and technical replicates following the protocol mentioned above. Expression of *msp-18* and *msp-20* in different transgenic lines were depicted as average ΔCt, i.e., difference between the Ct mean of transgene and the normalizer gene, *18S rRNA* (Gantasala et al., 2013; Papolu et al., 2016).

For detection of *msp-18* and *msp-20* siRNAs in T_1_ events, Northern analysis was performed. Small RNAs were extracted from the fresh leaves of PCR-positive T_1_ plants using a miRNA isolation kit (mirVana^TM^, Ambion) following the manufacturer’s instructions. Small RNAs (10 μg) of each event were electrophoresed on 15% denaturing PAGE gel followed by transblotting to a nitrocellulose membrane (Bio-Rad). Probes for *msp-18* and *msp-20* were labeled through PCR using digoxigenin (DIG) probe synthesis system (Roche Diagnostics). Blot hybridization and development were carried out according to the manufacturer’s instructions (Roche Diagnostics).

### Identification of Transgene Integration Loci in T_2_ Eggplants

In order to evaluate the T-DNA integration sites in transformed plants, *msp-18* and *msp-20* integration and flanking sequences in selected T_2_ events were identified using genome walking technique (Universal genome walker 2.0, Clontech laboratories). Genomic DNA was isolated from fresh leaves of T_2_ plants as described above. Two microgram DNA was used as the template in PCR reaction with T-DNA- and adapter-specific primers (enlisted in Supplementary Table S1) following the manufacturer’s instructions. The final PCR products were cloned and sequenced as described above. Sequences were analysed via NCBI-BLAST and sol genomic network^3^. In addition, event-specific PCR was performed to authenticate the specific site of integration in the progeny plants. The gene-specific and flanking primers are given in Supplementary Table S1.

### Bioefficacy Analysis of Transgenic Eggplants against *M. incognita*

Homozygous T_1_ plants harboring the RNAi constructs of *msp-18* and *msp-20* were subjected to bioefficacy studies against *M. incognita*. The roots of a fortnight-old T_1_ plants (growing in 250 ml pots containing equal mixture of soil and soilrite) were inoculated with approximately 300 J2s of *M. incognita* in the vicinity of the root tip. Nematode infected plants were grown at 28°C, 70% relative humidity and 16:8 h light:dark photoperiod in confinement. Plants were harvested at 30 dpi (days post inoculation). Different agronomic characters (root and shoot weight) of plants and nematode parasitic success on these plants [in terms of number of galls, egg masses, eggs/egg mass and multiplication factor (MF)] were recorded as described by Papolu et al. (2013, 2016) and Dutta et al. (2015b). The experiment was conducted twice with at least six replicates.

At 2 and 7 dpi, some of the plants from each treatment were harvested and roots were stained with acid fuchsin (Byrd et al., 1983) in order to estimate the nematode penetration ability on transgenic plants.

### Expression Analysis of *msp-18* and *msp-20* in Nematodes Extracted from Transgenic Eggplants

Total RNA was isolated from feeding females and reverse transcribed to cDNA as described above. qRT-PCR was performed and fold change in expression of target gene was calculated as explained above.

Additionally, the effect of HIGS on early parasitic J2s of *M. incognita* was analyzed. Seven-day-old transgenic (T_1_) and wild-type seedlings were placed independently at the center of a 50 mm Petri dish containing 5 ml of 23% pluronic gel (PF-127, Sigma; Dutta et al., 2011; Kumari et al., 2016) and approximately 100 J2s of *M. incognita* were inoculated behind the root tip of each plant. Plants were harvested at 8 h post inoculation because maximum numbers of J2s were latched on to the root tip during that time point. J2s clinging around the root tip were carefully extracted under the microscope, collected in a microcentrifuge tube and rinsed with sterile water for several times to remove the traces of PF-127. RNA was extracted from J2s and qRT-PCR assay was conducted as described above.

### Carbon Isotope (^14^CO_2_) Labeling of Wild-Type Eggplants

Seeds of wild-type eggplant (cv. Pusa Purple Long) were surface sterilized with 0.1% HgCl_2_ prior to sowing on a sterilized plastic bowl containing quartz sand in a growth chamber. At 7 days post germination of eggplant, bowls (each containing 70–80 seedlings) were transferred into an air tight Plexiglas chamber and exposed to ^14^CO_2_ which was released due to acidification of sodium ^14^C-bicarbonate (specific activity – 2.1 mCi/mmole) by 1 N HCl (Supplementary Figure S3). The plants were incubated in ^14^CO_2_ environment for 30 min followed by the pouring of KOH solution into the source Petri dish via PVC tubing to remove any unused ^14^CO_2_ in the chamber. After another 30 min, bowls containing ^14^CO_2_-labeled plants were removed from the chamber and transferred to the laboratory condition at room temperature. Detailed procedure is described in Pandey et al. (2013) and Singh et al. (2014).

### Autoradiography of Plant Material

In order to visualize the transport of carbon within the labeled plant, autoradiogram analysis was done. The plants were removed gently from the bowl and washed with deionized water to remove any external contaminants. Wrapped in filter paper plants were instantly dried at 80°C in a gel drier for 20 min. The plants were further flattened under a weight for 5 days. Subsequently, the plants were wrapped in transparent cling film and exposed to Fujifilm (Kodak) for 1 week.

### Liquid Scintillation Counting in Plant Material and Penetrating Nematodes

The incorporation of ^14^C into the different metabolic pools (i.e., roots and shoots) of eggplant was determined by measuring an aliquot of the respective fraction using a liquid scintillation counter (TRICARB, Perkin Elmer). The labeled plants were dried at 60°C in an oven for overnight followed by crushing of the roots and shoots using sterilized pestle and mortar. Twenty milligram of Cab-O-Sil (stabilizes sample suspension by thixotropy, Cabot Corp. Inc.) and 4 ml of Ultima Gold Scintillation Cocktail (Perkin Elmer) were added to the known volume of the dried sample, incubated for 30 min in dark and scintillation counts were measured.

In parallel, the fresh labeled plants were put in the PF-127 medium in a Petri dish (each containing five plants; Supplementary Figure S3) and challenge inoculated with 100 J2s of *M. incognita* as described above. At 8 h post inoculation, J2s were gently separated from the root tip and washed with deionized water for at least 20 times in order to remove external contaminants. Afterward, J2s were pooled together in an eppendorf tube and dried *in vacuo*. The known volume of the dry residue was dissolved in Cab-O-Sil and scintillation liquid cocktail, and scintillation counts were measured. The total ^14^C assimilation was calculated as disintegrations per minute (dpm) per mg dry weight. The relative ^14^C assimilation was expressed as percentage in shoot, root, and nematodes separately.

### Statistical Analysis

Bioassay and expression data were analyzed by one-way ANOVA and Tukey’s HSD test (significance level at *P* < 0.05) using SAS version 9.3 for Windows (SAS Software, Inc.).

## Results

### Stage-Specific Expression of CWME and Pioneer Gene Transcripts in Different Developmental Stages of *M. incognita*

**FIGURE 1:**
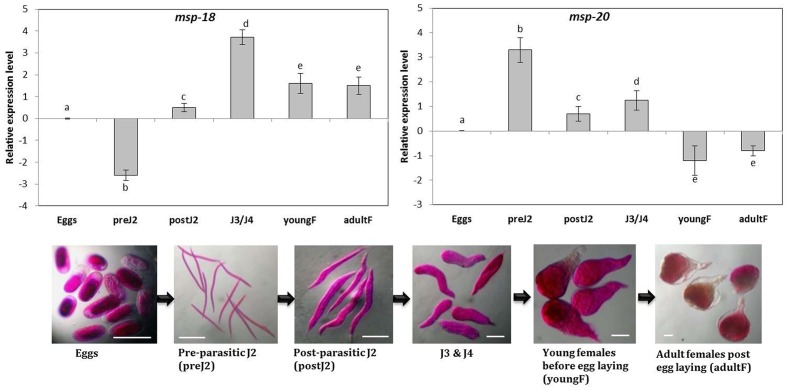
**Relative transcript abundance of *msp-18* and *msp-20* genes in different developmental stages of *Meloidogyne incognita***. Using the transcript level in eggs as reference, candidate genes (expression level was quantified by 2^-ΔΔCt^ method) were significantly upregulated or downregulated or unaltered in different life stages. Each bar represents the log_2_-transformed mean of quantitative reverse-transcription PCR (qRT-PCR) runs in triplicate with standard errors. Letters indicate significant differences using Tukey’s HSD test (*P* < 0.05). Scale bar = 100 μm.

Sequencing analysis of the cloned fragments of *msp-18* (456 bp), *msp-20* (598 bp), *Mi-xyl-1* (610 bp), *Mi-xyl-3* (610 bp), *Mi-pg-1* (547 bp), *Mi-eng-1* (585 bp), and *Mi-pel* (452 bp) revealed 100% identity to the reported sequences of *M. incognita*. Simultaneously, comparative expression of these genes in six developmental stages of *M. incognita* was carried out through qRT-PCR. Using the expression level in eggs as reference, mRNA levels of *msp-18* were significantly (*P* < 0.05) downregulated in pre-parasitic J2 in contrast to consistent upregulation in post-parasitic J2, J3/J4 mixed stages, young and adult females (**Figure 1**). Conversely, *msp-20* was significantly (*P* < 0.05) overexpressed in pre- and post-parasitic J2s compared to its marked repression in young and mature females (**Figure 1**). Transcripts of all the selected CWMEs were considerably upregulated in the early parasitic stages of *M. incognita* compared to their marked downregulation in young and egg laying females (Supplementary Figure S4). Taken together, qRT-PCR data demonstrates the association of *msp-20* and *msp-18* with the early and late stage of RKN parasitism in eggplant. Role of CWMEs in early parasitic stage of RKN was also re-established.

### Molecular Characterization of Transgenic Eggplant Harboring HIGS Constructs of *msp-18* and *msp-20*

Using the leaf disk*-Agrobacterium* co-cultivation method *A. tumefaciens* (LBA4404) harboring the HIGS constructs of *msp-18* and *msp-20* were used to transform eggplant (Supplementary Figures S1, S2). After kanamycin selection and regeneration, 25 primary transgenic events (T_0_) for each gene were generated. Each event was genotyped through PCR and presence of gene-specific, sense, antisense, and selectable marker fragment was detected in 15 and 14 events for *msp-18* and *msp-20* genes, respectively (Supplementary Figure S5). No significant difference in the morphological characters of untransformed control plants grown on non-selective medium and T_0_ plants grown on selection medium was observed (data not shown), indicating that neither the antibiotic marker nor the HIGS construct expression affected the growth of transgenic plants.

**FIGURE 2:**
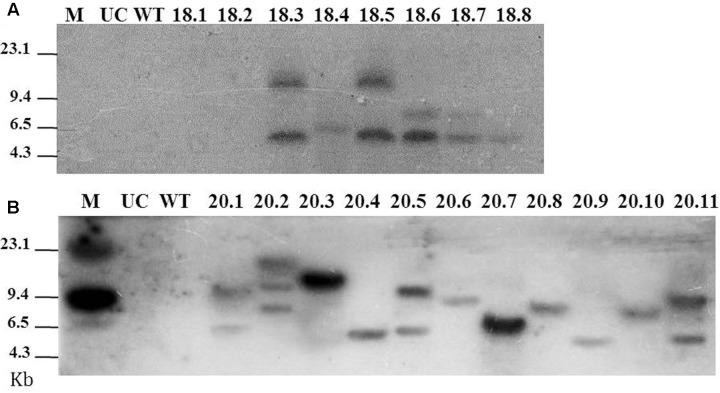
**Southern blot for T_0_ eggplant transformants harboring the Host-induced gene silencing (HIGS) construct of *msp-18* (A)** and *msp 20* **(B)** genes. Probes used for hybridization were specific to *msp-18* and *msp-20* genes. Untransformed control (UC) and wild-type (WT) plants did not show any hybridization signal to either of the probes. M: Lambda *Hind*III digest marker.

A total of 8 and 11 T_0_ lines were subjected to Southern blot assay to analyze the integration patterns of *msp-18* and *msp-20* transgenes, respectively (because in a preliminary assay hybridized fragments were detected only in these events). For *msp-18*, single copy insertion of T-DNA was observed in event numbers 18.4 and 18.8, while double copy insertion was documented for event numbers 18.3, 18.5, 18.6, and 18.7 (**Figure 2A**). For *msp-20*, single copy integration of the transgene was observed in event numbers 20.3, 20.4, 20.6, 20.7, 20.8, 20.9, and 20.10, while double copy integration was recorded in event numbers 20.1, 20.5, and 20.11 (**Figure 2B**). Henceforth, 6 and 10 events harboring the *msp-18* and *msp-20* transgenes, respectively, were used for further study.

T_1_ progeny plants were generated in the growth chamber by germinating the seeds of selected T_0_ events in the presence of antibiotic kanamycin. T_1_ plants were genotyped through PCR and expected fragments were detected (Supplementary Figure S6). This indicates the stable integration and inheritance of *msp-18* and *msp-20* RNAi transgenes in the progeny plants.

### Expression Analysis of *msp-18* and *msp-20* in T_1_ Eggplants

**FIGURE 3:**
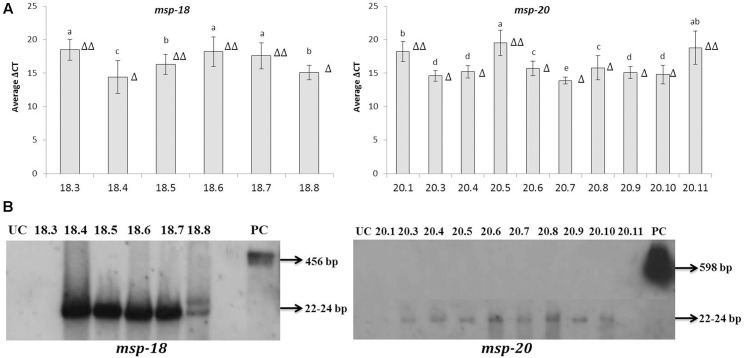
**Detection of *msp-18* and *msp-20* expression in T_1_ eggplant transformants by qRT-PCR and Northern blotting**. **(A)** Relative transcript levels of *msp-18* and *msp-20* are expressed as ΔCt values which denote the difference in Ct mean of transgene and the reference gene (*18S rRNA* gene of eggplant). Higher values represent the lower expression of the transgene. Each bar represents mean ± SEM derived from three independent biological and three technical replicates. Bars with different letters are statistically different at *P* < 0.05. **(B)** Blots showing siRNAs (typically 22–24 bp long) specific to *msp-18* and *msp-20* genes in different events. As positive control (PC) blots were independently probed with *msp-18* (456 bp) and *msp-20* (598 bp) genes. UC plants did not show any hybridization signal. Number of triangle (Δ) indicates the number of gene copies in each event.

Quantitative reverse-transcription PCR assay indicated the overexpression of *msp-18* and *msp-20* mRNAs in all the selected T_1_ events. By contrast, no transcripts corresponding to either of *msp-18* or *msp-20* were detected in the RNA extracted from untransformed control plants. Hence, the expression data of *msp-18* and *msp-20* for the transformed lines are simply presented (as ΔCt) relative to the normalizer gene, *18S rRNA*. Since Ct value is inversely proportional to expression quantification, significantly (*P* < 0.05) greater quantitative expression of *msp-18* was documented in event numbers 18.4, 18.8 (single copy events), and 18.5 compared to event numbers 18.3, 18.6, and 18.7. Likewise, comparatively greater induction of *msp-20* was recorded in single copy events (20.3, 20.4, 20.6, 20.7, 20.8, 20.9, and 20.10) than the double copy ones (20.1, 20.5, and 20.11) (**Figure 3A**).

As an integral component of HIGS, expression of *msp-18* and *msp-20* siRNAs in selected transgenic lines was demonstrated by Northern analysis. SiRNAs specific to *msp-18* gene were detected in both single (18.4 and 18.8) and double copy (18.5, 18.6, and 18.7) events. Similarly, *msp-20* siRNAs were detected in both single (20.3, 20.4, 20.6, 20.7, 20.8, 20.9, and 20.10) and double copy (20.5) events. However, RNAs isolated from event numbers 18.3 (*msp-18*-specific), 20.1 and 20.11 (*msp-20*-specific) did not show any hybridization signal with their respective probes (**Figure 3B**).

### Assessment of Transgene Integration Loci in T_2_ Eggplants

T_2_ progeny plants were generated from the selected T_1_ events through kanamycin selection. T_2_ plants were genotyped through PCR and expected fragments were detected (data not shown). In order to characterize the T-DNA integration sites in selected T_2_ events, event numbers 18.4 (*msp-18*-specific, single copy) and 20.7 (*msp-20*-specific, single copy) were evaluated using genome walking analysis because these events showed the greatest expression of their respective transgenes in qRT-PCR assays. Using event-specific primers fragments flanking the T-DNA region were detected in events 18.4 and 20.7 as well as in their progeny plants. The absence of these fragments in other *msp-18*- and *msp-20*-specific events confirmed the independent integration of both the selected events (Supplementary Figure S7). The T-DNA region flanking the sequences of both *msp-18* and *msp-20* transgenes showed homology with the eggplant genome sequences. The sequence of the flanking PCR product corresponding to event 18.4 showed a maximum of 97% identity with *Solanum pennellii* chromosome 1 (Sequence ID: HG975440). Whereas the sequence obtained from event 20.7 showed a maximum of 84% homology with the *S. melongena* draft genome sequence (Sequence ID: Sme2.5_00742.1).

### Evaluation of T_1_ and T_2_ Eggplants for Resistance against *M. incognita*

**FIGURE 4:**
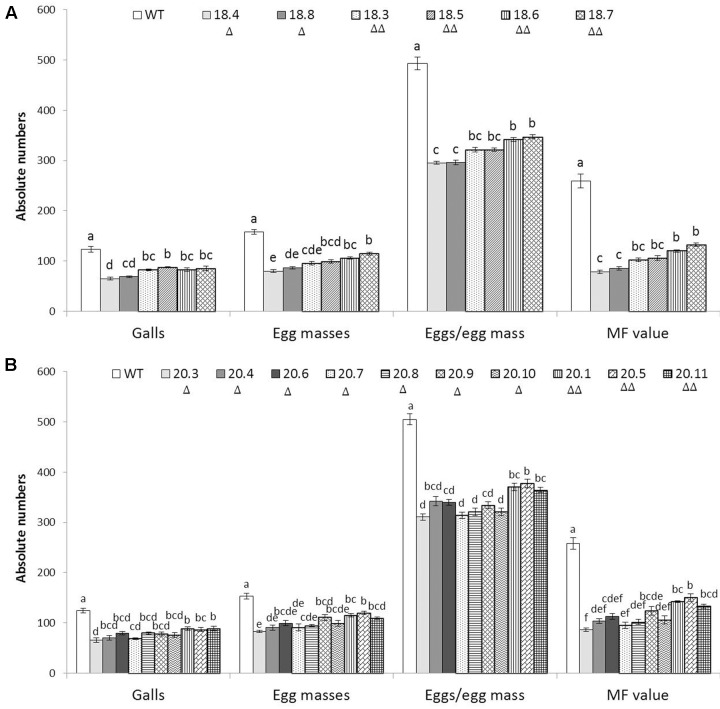
**Effect of HIGS of (A)** *msp-18* and **(B)** *msp-20* genes on development and reproduction of *M. incognita* in eggplant. Absolute numbers of galls, egg masses, eggs/egg mass, and the corresponding multiplication factor (MF) of *M. incognita* in different T_1_ events (*msp-18*-specific: 18.4, 18.8, 18.3, 18.5, 18.6, and 18.7; *msp-20*-specific: 20.3, 20.4, 20.6, 20.7, 20.8, 20.9, 20.10, 20.1, 20.5, and 20.11) and WT plants at 30 dpi (days post inoculation). Each bar represents the mean ± SE of *n* = 6, and bars with different letters (within each parameter) denote a significant difference at *P* < 0.05, Tukey’s test. Number of triangle (Δ) indicates the number of gene copies in each event.

To assess whether HIGS of two target genes confers resistance to *M. incognita*, 6 and 10 independent T_1_ events harboring the RNAi constructs of *msp-18* and *msp-20*, respectively, were evaluated in terms of nematode invasion, development and reproduction in the host roots. At 30 dpi, number of root galls was considerably reduced in different T_1_ events compared to wild-type plants. Additionally, T_1_ plants have showed more robust root system than the wild-type plants (Supplementary Figure S8). The average number of galls per plant was significantly (*P* < 0.05) reduced by 30.81–47.29% and 28.22–46.77% in *msp-18* and *msp-20* RNAi lines, respectively, compared to the wild-type. Accordingly, a marked reduction in the number of egg masses (27.27–49.25% for *msp-18* and 22.17–45.86% for *msp-20*) was recorded in all the events compared to wild-type ones (**Figure 4** and Supplementary Table S2). Because each female produces its progeny in a single egg mass, the number of egg masses indicates the identical number of successfully reproducing females. This suggests that the HIGS of *msp-18* and *msp-20* genes resulted in the reduced root galling due to impeded developmental progression of *M. incognita* juveniles to females in transgenic plants. In corroboration, fecundity of *M. incognita* in terms of number of eggs per egg mass was substantially decreased by 29.66–40.20% and 25.21–38.48% in *msp-18* and *msp-20* RNAi lines, respectively, compared to wild-type plants (**Figure 4** and Supplementary Table S2). Considering the importance of MF as determinant of nematode reproductive fitness and parasitic success on a host plant, our results demonstrated that MF was significantly (*P* < 0.05) decreased by 48.93–69.68% and 41.74–66.69% in *msp-18* and *msp-20* RNAi lines, respectively, compared to wild-type plants. Among *msp-18* RNAi lines, single copy transformants (18.4, 18.8) exhibited significantly (*P* < 0.05) greater reduction in MF than some of the double copy transformants (18.6, 18.7). Similarly, in case of *msp-20* RNAi lines, some of the single copy transformants (20.3, 20.4, 20.7, 20.8, 20.10) showed significantly (*P* < 0.05) greater reduction in MF than the double copy ones (20.1, 20.5, 20.11). Interestingly, within the single or double copy transformants no significant difference (*P* < 0.05) could be observed (**Figure 4** and Supplementary Table S2). Comparable results were obtained when T_2_ plants of both *msp-18* and *msp-20* RNAi lines were tested for their bioefficacy against *M. incognita* (Supplementary Figure S9 and Table S2) suggesting that there was no resistance breakdown in the progeny plants. The complete experiment was carried out twice and showed similar results on each occasion.

**FIGURE 5:**
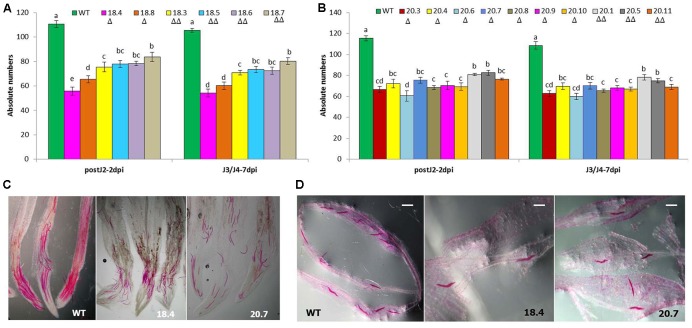
**Comparative invasion and development of *M. incognita* in WT and T_1_ (*msp-18*-specific: 18.4, 18.8, 18.3, 18.5, 18.6, and 18.7; *msp-20*-specific: 20.3, 20.4, 20.6, 20.7, 20.8, 20.9, 20.10, 20.1, 20.5, and 20.11) events of eggplant at (A)** 2 dpi (as post-parasitic J2) and **(B)** 7 dpi (as spike-tail J3 and J4). Each bar represents the mean ± SE of *n* = 3, and bars with different letters denote a significant difference at *P* < 0.05, Tukey’s test. Photographs showing the stained post-parasitic J2s **(C)** and spike-tail stages **(D)** in the roots of events 18.4 and 20.7, and WT. Nematodes were stained with acid fuchsin. Scale bar = 500 μm. Number of triangle (Δ) indicates the number of gene copies in each event.

In addition, perturbed penetration ability of *M. incognita* J2 at 2 dpi and consequently, significantly (*P* < 0.05) less number of spike-tail stages (J3/J4) at 7 dpi was documented in the T_1_ plants compared to wild-type ones (**Figure 5**). This indicated the detrimental effect of HIGS of *msp-18* and *msp-20* genes on the early parasitic stages of *M. incognita*.

### Expression Analysis of CWME and Pioneer Genes in Nematodes Extracted from Transgenic Eggplants

**FIGURE 6:**
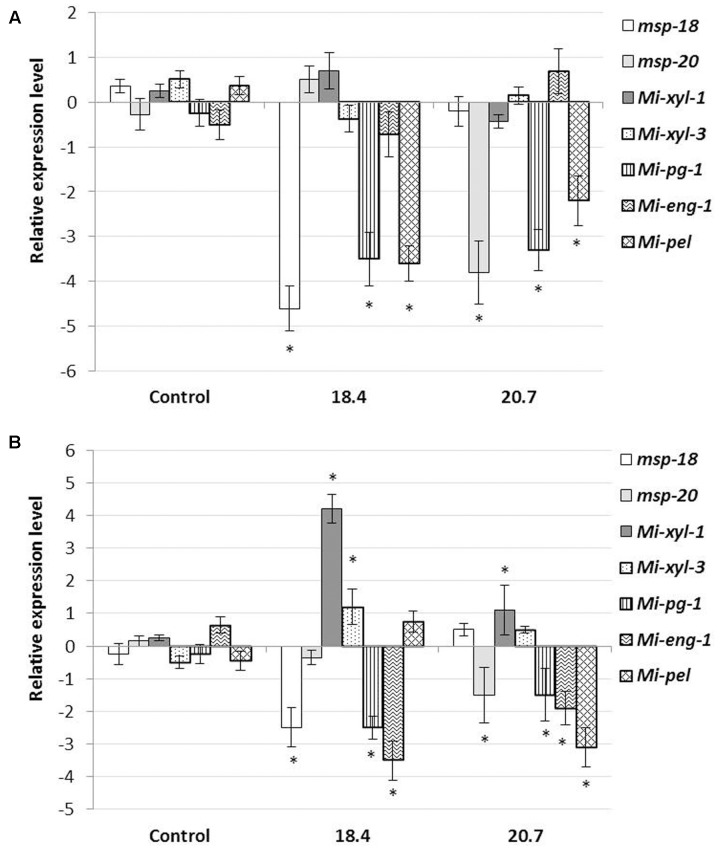
**Effect of HIGS on the transcript abundance of cell-wall modifying enzyme (CWME) and pioneer genes in feeding females (A)** and early parasitic J2s **(B)** of *M. incognita* extracted from the roots of T_1_ events (18.4 and 20.7) at 20 dpi and 8 h post inoculation, respectively. Using *18S rRNA* gene as reference, expression was quantified via 2^-ΔΔCt^ method and fold change values were log_2_-transformed. Bars represent mean expression levels and standard error from three biological and three technical replicates. Asterisks indicate significant differential expression (*P <* 0.05) in comparison with the nematodes extracted from the non-transgenic control plants.

In order to investigate the effect of HIGS on the effector gene abundance in nematodes, qRT-PCR was performed to analyse the possible change in transcript level of CWME (*Mi-xyl-1*, *Mi-xyl-3*, *Mi-pg-1*, *Mi-eng-1*, and *Mi-pel*) and pioneer genes (*msp-18* and *msp-20*) in *M. incognita* females extracted from the roots of T_1_ events (18.4 and 20.7) at 20 dpi (event 18.4 and 20.7 were found to be the best transgenic lines in suppressing RKN parasitic ability). Results showed that the mRNA levels of *msp-18* and *msp-20* were significantly (*P* < 0.05) downregulated in events 18.4 and 20.7, respectively, compared to females isolated from non-transgenic control plants (**Figure 6A**). Expectedly, no significant transcriptional alteration was documented for *msp-18* and *msp-20* in events 20.7 and 18.4, respectively, suggesting the target-specific silencing effect of pioneer genes. Intriguingly, HIGS of pioneer genes had led to the significant (*P* < 0.05) downregulation of CWME genes (*Mi-pg-1* and *Mi-pel*) in females developing in the events 18.4 and 20.7, compared to control (**Figure 6A**).

To further confirm the possibility of effector crosstalk among pioneer and CWME genes, qRT-PCR was performed with the early parasitic J2s of *M. incognita* extracted from the root tip of T_1_ plants (18.4 and 20.7) at 8 h post inoculation (in PF-127 medium). Significant (*P* < 0.05) repression of *msp-18* and *msp-20* genes in J2s extracted from the events 18.4 and 20.7, respectively, and their non-significant expression vice versa (**Figure 6B**), have exemplified the target-specific silencing effect of pioneer genes. Concomitantly, significant (*P* < 0.05) overexpression of *Mi-xyl-1* and *Mi-xyl-3*, and repression of *Mi-pg-1* and *Mi-eng-1* was documented in J2s infecting plants (18.4) that express *msp-18* siRNAs. Similarly, *Mi-xyl-1* was significantly (*P* < 0.05) overexpressed and *Mi-pg-1*, *Mi-eng-1*, and *Mi-pel* were downregulated in J2s isolated from event 20.7 that expresses *msp-20* siRNAs (**Figure 6B**). Taken together, these data reveal that the dsRNA/siRNA molecules corresponding to *msp-18*/*msp-20* genes were ingested by the nematodes as early as during the process of penetration.

### Detection of Carbon Export in *M. incognita* J2 Infecting the ^14^CO_2_-Labeled Eggplant

**FIGURE 7:**
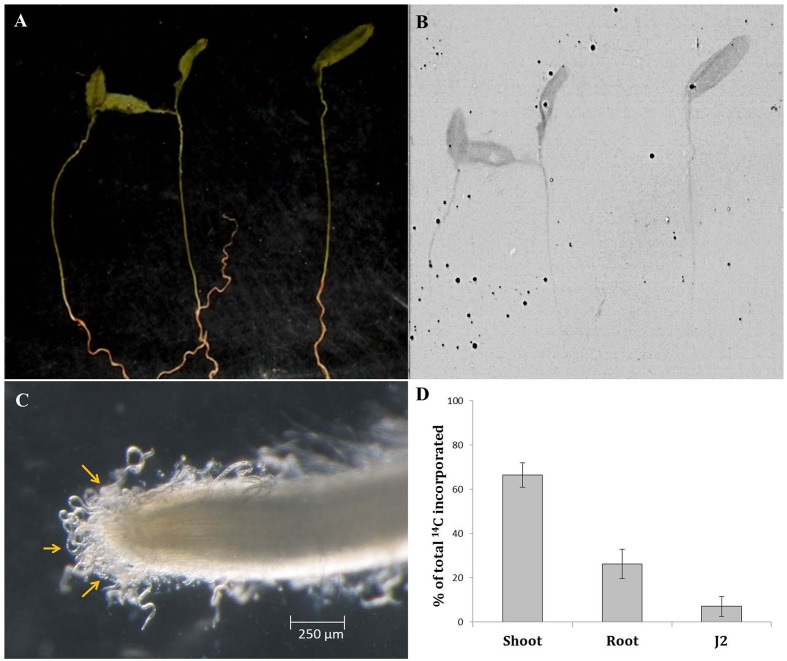
**Qualitative and quantitative representation of carbon transport from source to sink tissues**. Picture **(A)** and autoradiogram **(B)** of 7-day-old eggplants (cv. Pusa Purple Long) labeled with ^14^CO_2_. Plants were labeled for 30 min in an airtight chamber followed by a chase period of 2 h in air. Dried plant material was exposed to the film for 7 days. **(C)** Infective *M. incognita* J2s latching on to the root tip of a labeled plant at 8 h post inoculation. One hundred J2s were inoculated behind the root tip in pluronic gel medium. **(D)** Relative ^14^C assimilation in shoot, root, and penetrating J2. The sum of the label is set to 100%. Mean ± SE (*n* = 12). Liquid scintillation counts (as dpm) of unlabelled plant material and freshly hatched J2s were used as the control.

In order to validate the hypothesis that nematodes may ingest host-derived molecules during the earliest phase of plant–nematode interaction (i.e., prior to initiation of feeding cell), wild-type eggplants were radiolabelled with carbon isotope (^14^CO_2_) and inoculated with *M. incognita* J2 in PF-127 medium for 8 h followed by the scintillation counting of ^14^C in penetrating J2s. The autoradiographs revealed that ^14^C was incorporated throughout the plant tissue indicating the successful labeling of the plant material (**Figures 7A,B**). Quantitative measurements based on liquid scintillation counts (after deducting the dpm counts for unlabelled plant material and freshly hatched J2 as control) showed that 66.5, 26.3, and 7.2% of the total label was assimilated in the shoot, root, and J2s, respectively (**Figures 7C,D** and Supplementary Figure S10). The less intense radioactive signal in the root tissues can be explained by the possibility that a portion of the labeled carbon was lost during the course of the experiment due to respiration in heterotrophic tissues such as root. Nevertheless, detection of a significant proportion of the labeled carbon in J2 suggests that nematodes can ingest the cell sap containing plant-derived molecules while thrusting its stylet to enter into the plant tissue.

## Discussion

The parasitic success of *M. incognita* (arguably the most damaging phytonematode) in a broader range of host crops largely depends on the effective deployment of its repertoire of effector genes that facilitate host invasion, evade host defense responses and assists in establishing a feeding site. During the last two decades a lot of research was conducted to identify and functionally characterize those effector molecules using various means (Hassan et al., 2010; Haegeman et al., 2012; Mitchum et al., 2013), however, none of the studies have addressed the issues related to effector crosstalk in nematodes during successful parasitism of the host tissue. Earlier, we have detected the interaction among CWME and pioneer genes in *M. incognita* using *in vitro* and *in vivo* reverse-genetics approach (Shivakumara et al., 2016). In continuation of that study, here, using HIGS approach we have revalidated the effector–effector interaction among two pioneer and five CWME genes of *M. incognita* during penetration and development of the host plant such as eggplant. It is to be noted that in the absence of any suitable genetic transformation method, RNAi is a tractable tool to study the possible interaction among effector molecules in phytonematodes.

Initially, to investigate the differential expression of selected effectors throughout the developmental stage of RKN, qRT-PCR analysis was used. The transcript level of *msp-18* was found to be relatively higher in advanced life stages than the pre-parasitic J2 stage. On the contrary, expression of *msp-20* was comparatively greater in pre-parasitic J2 than the other life stages. All the CWMEs (*Mi-xyl-1*, *Mi-xyl-3*, *Mi-pg-1*, *Mi-eng-1*, and *Mi-pel*) were quantitatively greatly expressed in pre- and post-parasitic J2 stages. Afterward, eggplants were successfully transformed with RNAi constructs of *msp-18* and *msp-20* genes in order to evaluate their effect on *M. incognita* infectivity and transcription pattern of CWME genes in infecting nematodes. Six (18.3, 18.4, 18.5, 18.6, 18.7, and 18.8) and ten (20.1, 20.3, 20.4, 20.5, 20.6, 20.7, 20.8, 20.9, 20.10, and 20.11) transgenic events containing both single and double copy insertions of *msp-18* and *msp-20* transgenes, respectively, were selected for further studies. Interestingly, qRT-PCR analysis revealed significantly greater expression of both *msp-18* and *msp-20* transgenes in single copy T_1_ lines than the double copy ones. In corroboration, single copy T_1_ and T_2_ lines conferred significantly greater nematode resistance (in terms of reduction in *M. incognita* MF) than the double copy events for both *msp-18* and *msp-20* genes. The relatively lower resistance of double copy transformants to RKN can be explained by the likelihood of transgenes co-suppressing others in the vicinity in a homology-dependent manner. Similar silencing among transgenes and endogenous genes with a high level of homology was reported in several plant species (Napoli et al., 1990).

The T-DNA integration sites of *msp-18* and *msp-20* transgenes were characterized in selective T_2_ events, 18.4 and 20.7, respectively, and event-specific PCR failed to detect the expected fragments in events other than 18.4 and 20.7. This suggests that the transgenes were randomly integrated at various transcriptionally active sites in the eggplant genome which confirmed the independent nature of the selected events (This also confirmed the absence of the T-DNA integration site effect on the observed phenotypes).

Detection of target gene siRNAs of both *msp-18* and *msp-20* in their respective T_1_ events using Northern analysis provided the ultimate evidence for occurrence of HIGS which was complemented by the bioefficacy data against *M. incognita*. In addition, a pronounced reduction in nematode penetration and development (to J3/J4) was also documented in the transgenic plants. Although the transgenic delivery of dsRNA/siRNA of *msp-18* and *msp-20* genes via plant cells to *M. incognita* caused developmental and reproductive retardation in feeding nematodes, none of the eggplant lines exhibited complete nematode resistance. Our data is in agreement with the HIGS studies of other nematode parasitism genes (Dutta et al., 2015a) which showed partial nematode resistance. In order to increase the resistance level, combinatorial HIGS was attempted using fusion construct of cysteine and serine protease (*Mi-cpl-1*and *Mi-ser-1*) genes of *M. incognita* in tomato (Antonino de Souza Júnior et al., 2013). However, superior nematode resistance could not be achieved (compared to RNAi construct of either protease genes alone) in that study may be because high level of dsRNA delivery saturates the dsRNA processing ability of nematodes. On the contrary, *Arabidopsis thaliana* expressing compound dsRNA constructs of housekeeping genes due to crossing of different RNAi lines showed enhanced HIGS efficacy against *M. incognita* (Charlton et al., 2010). Nevertheless, achieving complete nematode resistance is practically impossible and 60% reduction in nematode multiplication per generation (RKNs complete three generations in an annual host crop) is sufficient to bring down nematode population below the threshold level (Ehwaeti et al., 2000; Fuller et al., 2008). It is to be noted that in our study, the transgenic events, 18.4 (*msp-18*-specific) and 20.3 (*msp-20*-specific) conferred 69.68 and 67.30% resistance to RKN, respectively, in terms of reduced MF.

In order to substantiate the long-term effect of HIGS on nematode development, females of *M. incognita* that developed on the selective T_1_ events, 18.4 and 20.7, expressing siRNAs of *msp-18* and *msp-20* genes, respectively, were extracted and subjected to qRT-PCR analysis. A significant reduction in *msp-18* and *msp-20* expression was recorded in females extracted from their corresponding transgenic events. Hence, it can be speculated that the HIGS effect of *msp-18* and *msp-20* genes in *M. incognita* is systemic and propagates upon uptake of host-delivered dsRNA/siRNA molecules via stylet to the entire body. Since J3 and J4s are considered to be the non-feeding stages (Jones et al., 2013), RNAi effect was transmitted across the different developmental stages of RKN and presumably offspring having defunct *msp-18*/*msp-20* gene was produced. The heritable nature of RNAi has previously been described in *Caenorhabditis elegans* (Grishok et al., 2000), *M. javanica* (Gleason et al., 2008), and *M. chitwoodi* (Dinh et al., 2014).

Despite the positive reports of RNAi-induced phenotypic variation in phytonematodes, it still remains inconclusive that the resulting phenotype is due to the silencing of a specific target gene (Lilley et al., 2012; Dutta et al., 2015a). Presuming that the nematode parasitism genes may have additive, combinatorial or redundant function at the plant-nematode interface, we have evaluated the HIGS effect of *msp-18* and *msp-20* effectors on the transcriptional oscillation of other unrelated effectors such as CWMEs in *M. incognita* using qRT-PCR in the current study. Interestingly, females extracted from the T_1_ events, 18.4 and 20.7 (expressing *msp-18* and *msp-20* siRNAs, respectively), showed significant downregulation of *Mi-pg-1* and *Mi-pel* genes compared to females developed on non-transgenic control plants. In addition, pre-parasitic J2s were exposed to the roots of T_1_ events 18.4 and 20.7 in PF-127 medium and at 8 h post inoculation J2s clinging on the root tip were collected and subjected to qRT-PCR analysis. Intriguingly, apart from downregulation of the target gene (*msp-18*), J2s extracted from event 18.4 showed significant overexpression (*Mi-xyl-1* and *Mi-xyl-3*) and repression (*Mi-pg-1* and *Mi-eng-1*) of CWME genes compared to J2s infecting the control plants. Similarly, J2s isolated from event 20.7 exhibited significant upregulation of *Mi-xyl-1* and downregulation of *Mi-pg-1*, *Mi-eng-1*, and *Mi-pel* genes along with the expected reduction in target (*msp-20*) mRNA levels. However, overexpression of some specific CWMEs was not sufficient to increase *M. incognita* infectivity in transgenic eggplants (with reference to bioefficacy data in both T_1_ and T_2_ plants). To the best of our knowledge, this is the first report of *in planta* effector suppression effect on expression of other unrelated effectors indicating a potential interaction among them during nematode penetration of the host tissue. The possibility of off-target effects can be excluded because all the targeted CWME genes are phylogenetically much distant than the *msp-18*/*msp-20* genes (described in Shivakumara et al., 2016). Any flaw in the experimental method may also be dismissed because temporal expression of all the effectors was unaltered in nematodes extracted from non-transgenic control plants. In an isolated study in fungal pathogen, *Fusarium verticillioides*, a potential link between effector *FSR-1* and CWMEs were established speculating that the coiled-coil motif present in *FSR-1* undergoes protein–protein interaction with CWMEs. In *M. incognita*, *msp-20* (used in our study), *msp-4*, *msp-9*, *msp-10*, *msp-26*, *msp-34*, and *msp-40* (Huang et al., 2003; Niu et al., 2016) are known to have coiled-coil motifs. In addition, a number of pioneer genes contain nuclear localization domains and exhibit transcriptional activation activity *in planta* (Huang et al., 2003; Quentin et al., 2013; Zhang et al., 2015). Based on our study, it is assumed that the interaction among nematode effectors leads to some sort of retrograde signaling in the nematode body, which in turn cause decreased transcriptional expression of CWMEs during the parasitic process of RKN.

Our finding about transcriptional alterations of pioneer and CWME genes in early parasitic J2s (before penetration) clinging on the root tip of transgenic plants gives rise to the speculation that nematodes may acquire plant-derived molecules during the attachment on the host surface via the root exudates. Our data is in stark contrast with the earlier assumption that host-derived RNA molecules may only become available to the nematodes via the feeding tube which is formed during the formation of a feeding cell (Bakhetia et al., 2005). In order to authenticate our claim, we have labeled the wild-type eggplants with ^14^C isotope using standard protocol (Kölling et al., 2013; Singh et al., 2014) followed by challenge infection of the labeled plants with *M. incognita* J2s in PF-127 medium. At 8 h post inoculation J2s clinging on the root tip were carefully extracted under the microscope without damaging the root surface and utmost care was taken to minimize any traces of radioactivity on nematode surface, as described in methodology. The significant proportion of ^14^C incorporation in infecting J2 indicated that plant-derived molecules may get entry into the nematode body during the exploratory probing phase of nematodes on the root surface. This is the first report demonstrating the rapid assimilation of host-derived carbon by the invading J2 and denotes the potential of such strategy to trace the fate of plant-derived molecules in phytonematodes.

## Conclusion

Our data demonstrates that HIGS of *msp-18*/*msp-20* genes in eggplant confers substantial resistance to *M. incognita* by inducing deleterious effects on RKN penetration, development and reproduction. Although *msp-18* and *msp-20* genes are member of pioneer gene family and knockdown effect of one member may be masked by other member, transcriptional repression of CWME genes in invading nematodes may invariably protect the plant against *M. incognita* infection for longer period of time. The HIGS strategy ensures the continual delivery of bioactive RNA species to feeding nematodes (Gheysen and Vanholme, 2007). As no foreign proteins are expressed *in planta* and non-target organisms are least affected due to HIGS (Atkinson et al., 2012), deployment of this strategy offers an attractive alternative to manage nematode infestation below the threshold level.

## Author Contributions

TS, SC, and DK performed all the experiments. PP and RS helped in molecular characterization of transgenic events. PB helped in data analysis. BS and KM helped in radioactivity assays. TD and UR conceived the experiments and wrote the MS.

## Conflict of Interest Statement

The authors declare that the research was conducted in the absence of any commercial or financial relationships that could be construed as a potential conflict of interest.

## Footnotes

1 http://www.idtdna.com/calc/analyzer

2 http://www.psb.ugent.be/

3 http://solgenomics.net/
