# Supplementary material for: Host-Induced Silencing of Two Pharyngeal Gland Genes Conferred Transcriptional Alteration of Cell Wall-Modifying Enzymes of Meloidogyne incognita vis-à-vis Perturbed Nematode Infectivity in Eggplant
Source: Front Plant Sci. 2017 Mar 30;8:473. doi: 10.3389/fpls.2017.00473 (PMC5371666; doi:10.3389/fpls.2017.00473)
Supplement: Supplementary file 1 [file Presentation_1.PDF]

Supporting Information

Host-induced silencing of two pharyngeal gland genes conferred transcriptional alteration of cell wall-modifying enzymes of *Meloidogyne incognita vis-à-vis* perturbed nematode infectivity in eggplant

Tagginahalli N. Shivakumara[#1], Sonam Chaudhary[#1], Divya Kamaraju[#1], Tushar Kanti Dutta[1]*, Pradeep Kumar Papolu[1], Prakash Banakar[1], Rohini Sreevathsa[2], Bhupinder Singh[3], K.M. Manjaiah[4] and Uma Rao[1]*

Fig. S1. Development of RNAi constructs of *msp-18* and *msp-20* for *in planta* validation.

Fig. S2. Transformation of eggplant (cv. Pusa Purple Long) with RNAi construct and generation of transgenic lines.

Fig. S3. Experimental pipeline for conducting carbon isotope labelling and detection of radioactive carbon in eggplants.

Fig. S4. Relative transcript abundance of CWME genes in different developmental stages of *M. incognita*.

Fig. S5. PCR confirmation of RNAi constructs of *msp-18* (A) and *msp-20* (B) genes in eggplant transgenic events (T_0).

Fig. S6. PCR confirmation of RNAi constructs of *msp-18* (A) and *msp-20* (B) genes in eggplant transgenic events (T_1).

Fig. S7. Analysis of T-DNA integration sites in the genome of T_2 eggplant events 18.4 (A) and 20.7 (B) expressing HIGS constructs of *msp-18* and *msp-20*, respectively.

Fig. S8. Bioefficacy analysis of T_1 eggplants against *M. incognita*.

Fig. S9. Effect of HIGS of *msp-18* and *msp-20* genes on development and reproduction of *M. incognita* in eggplant.

Fig. S10. Calculation sheet of ^{14}C incorporation in shoot, root and infecting J2s in an average plant material.

Table S1 Percentage reduction in different parameters of *M. incognita* development and reproduction on eggplant events expressing HIGS constructs of *msp-18* and *msp-20* genes compared to the wild-type plants.

Table S2 List of primers used in the current study.

Figure S1. Development of RNAi constructs of *msp-18* and *msp-20* for *in planta* validation. The modular binary destination vector pK7GWIWG2(I) is schematically represented. The T-DNA portion contained *msp-18*/*msp-20* gene flanked by attR1 and attR2 site in one gateway cassette and their reverse complement in another gateway cassette, separated by an intron under the control of promoter CaMV 35S. When the construct is expressed in plants, an intron spliced out hairpin RNA (ihpRNA) of *msp-18*/*msp-20* is produced.

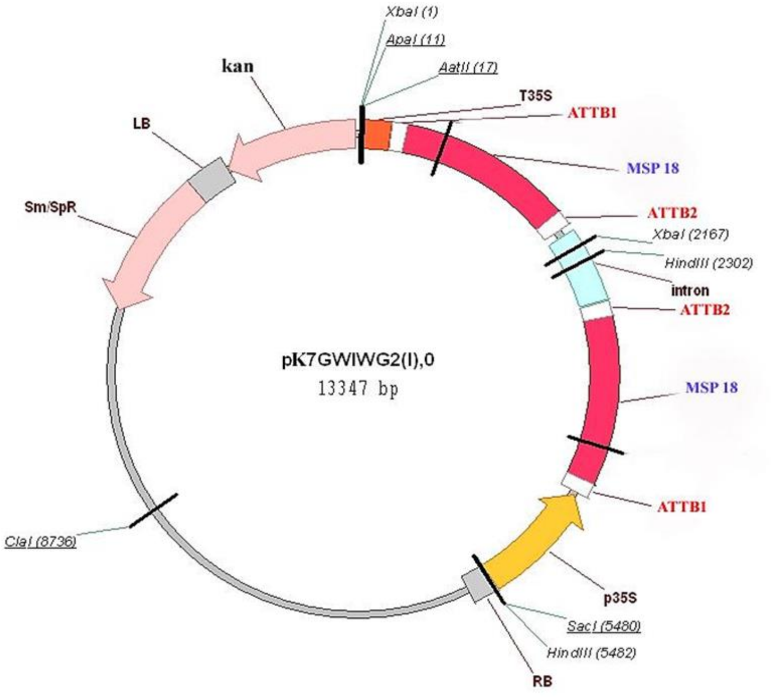

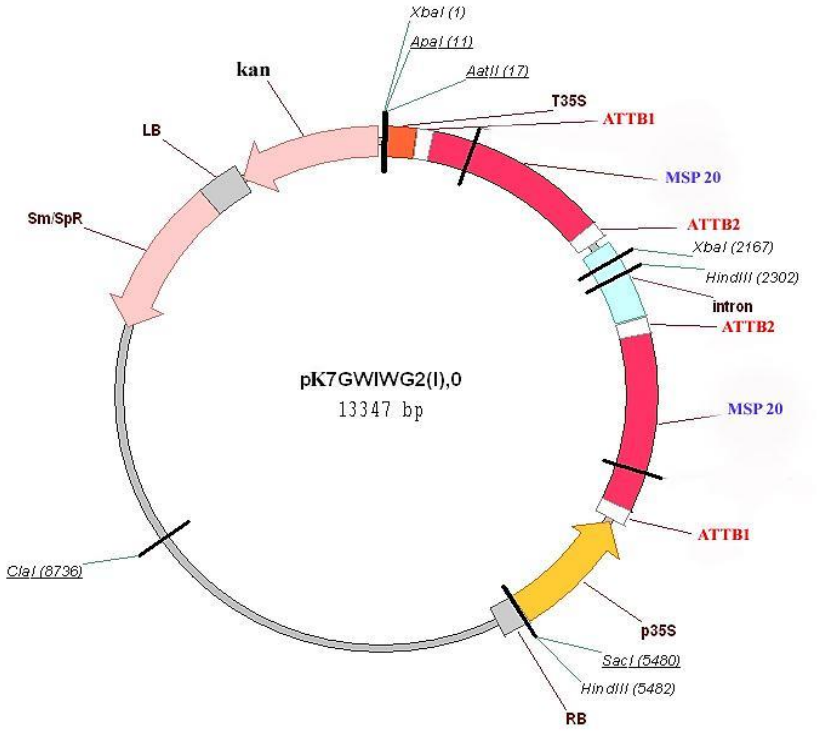

Figure S2. Transformation of eggplant (cv. Pusa Purple Long) with RNAi construct and generation of transgenic lines.

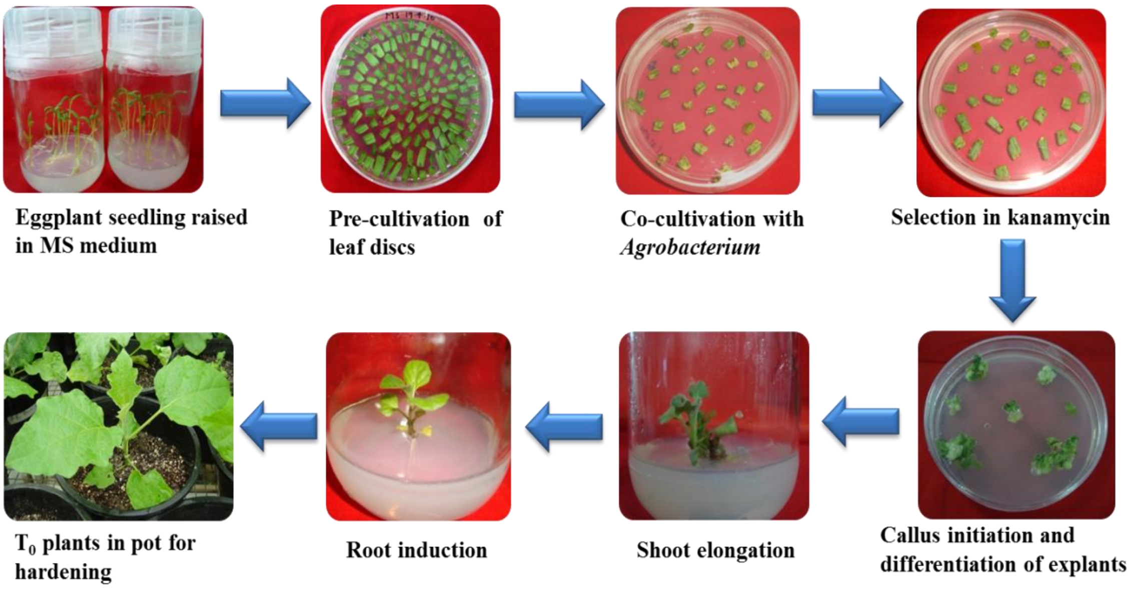

Figure S3. Experimental pipeline for conducting carbon isotope labelling and detection of radioactive carbon in eggplants. (A) Plastic bowls containing 7-day-old wild-type plants. (B) Plants in an air tight Plexiglas incubation chamber are exposed to $^{14}CO_2$ released by pouring 1 N HCl through the PVC tubing lowered in the chamber into the glass Petri plate containing $NaH^{14}CO_3$. (C) Radiolabeled plants are being put in the 5 cm Petri plate containing pluronic gel for nematode infection.

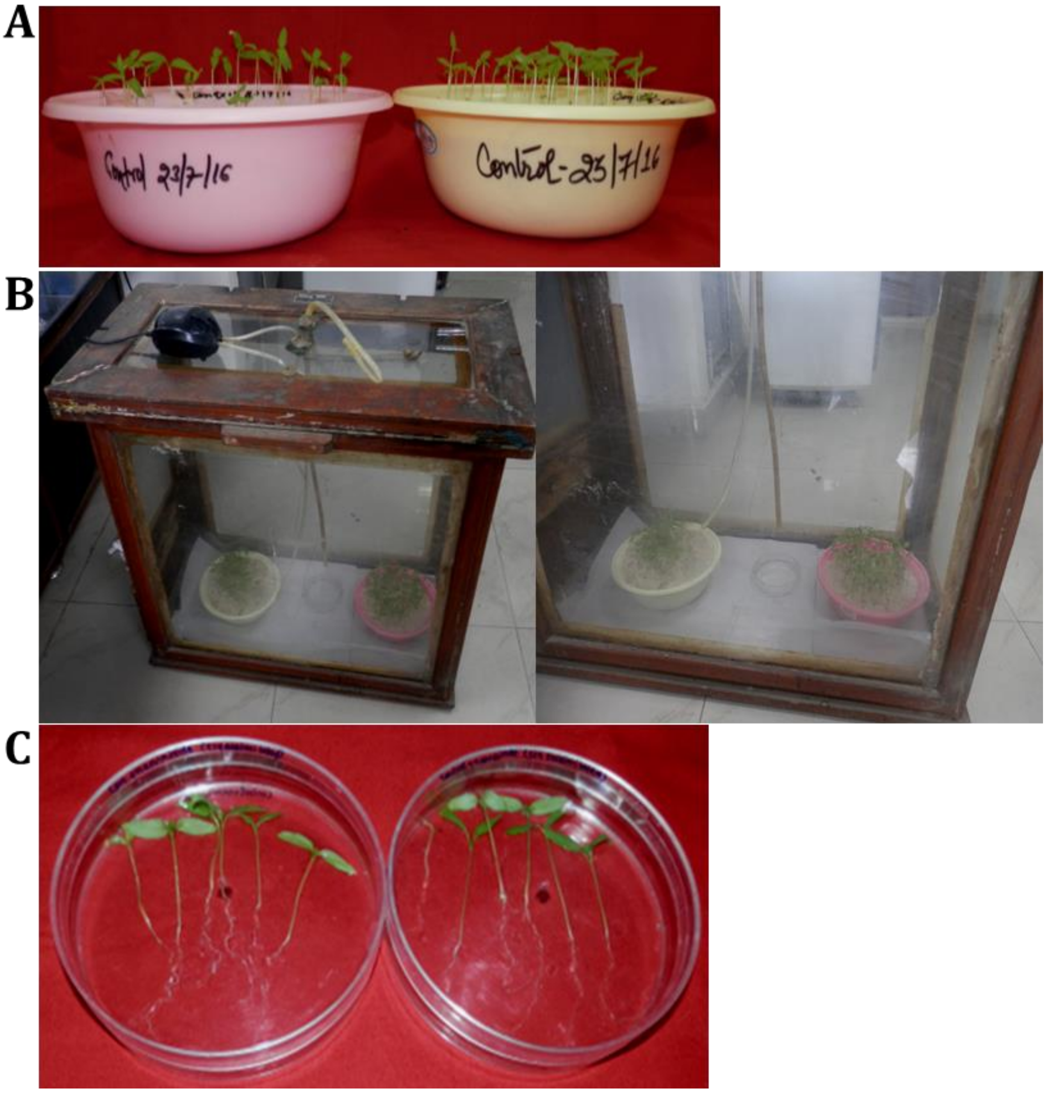

Figure S4. Relative transcript abundance of CWME genes in different developmental stages of *M. incognita*. Using the transcript level in eggs as reference, candidate genes (expression level was quantified by $2^{-\Delta\Delta Ct}$ method) were significantly upregulated or downregulated or unaltered in different life stages. Each bar represents the $\log_2$ transformed mean of qRT-PCR runs in triplicate with standard errors. Letters indicate significant differences using Tukey's HSD test (P<0.05).

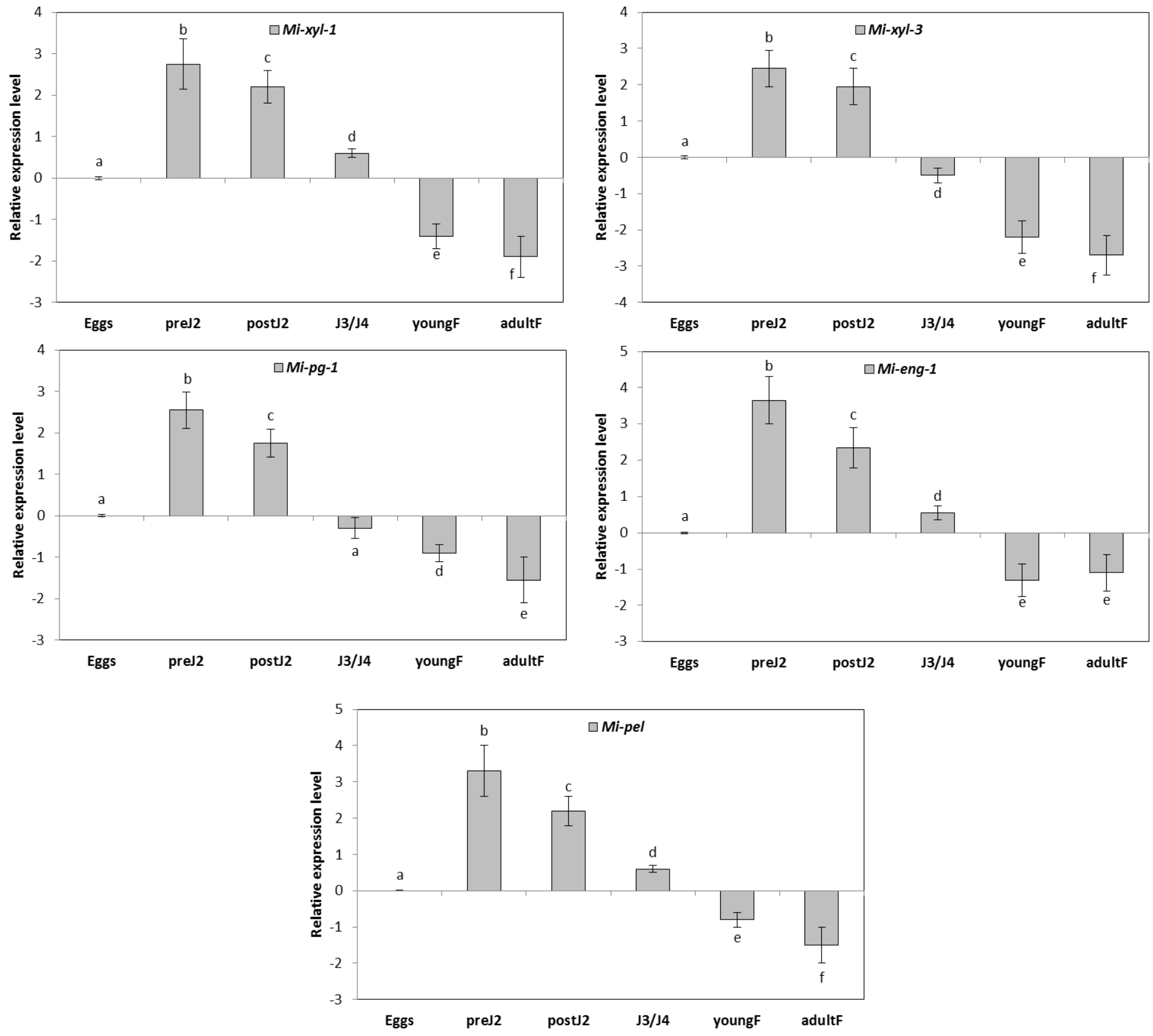

Figure S5. PCR confirmation of RNAi constructs of *msp-18* (A) and *msp-20* (B) genes in eggplant transgenic events (T_0). i) Amplification of the sense strand using primers 35S promoter forward and *attB2 reverse* (703 bp for *msp-18*, 789 bp for *msp-20*). ii) Amplification of the antisense strand using primers 35S terminator forward and *attB2 reverse* (673 bp for *msp-18*, 759 bp for *msp-20*). iii) Amplification of the target genes flanked by attB sites using gene specific primers (515 bp for *msp-18*, 659 bp for *msp-20*). iv) Amplification of the antibiotic marker gene, *nptII* (750 bp). Lanes - M: 100 bp DNA Ladder, +C – positive control, 1-15: T_0 events, UC – untransformed control.

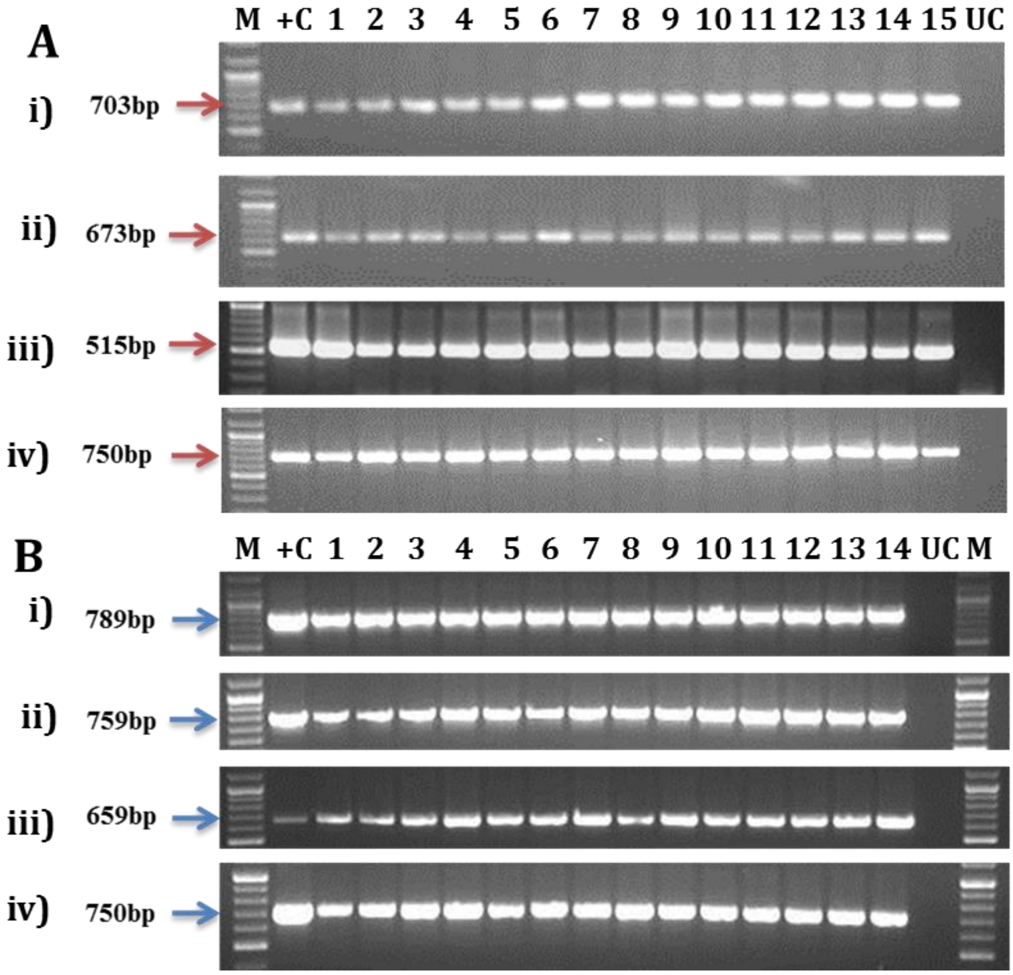

Figure S6. PCR confirmation of RNAi constructs of *msp-18* (A) and *msp-20* (B) genes in eggplant transgenic events (T_1). i) Amplification of the sense strand using primers 35S promoter forward and *attB2 reverse* (703 bp for *msp-18*, 789 bp for *msp-20*). ii) Amplification of the antisense strand using primers 35S terminator forward and *attB2 reverse* (673 bp for *msp-18*, 759 bp for *msp-20*). iii) Amplification of the target genes flanked by attB sites using gene specific primers (515 bp for *msp-18*, 659 bp for *msp-20*). iv) Amplification of the antibiotic marker gene, *nptII* (750 bp). Lanes - M: 100 bp DNA Ladder, +C – positive control, 1-15: T_1 events, UC – untransformed control.

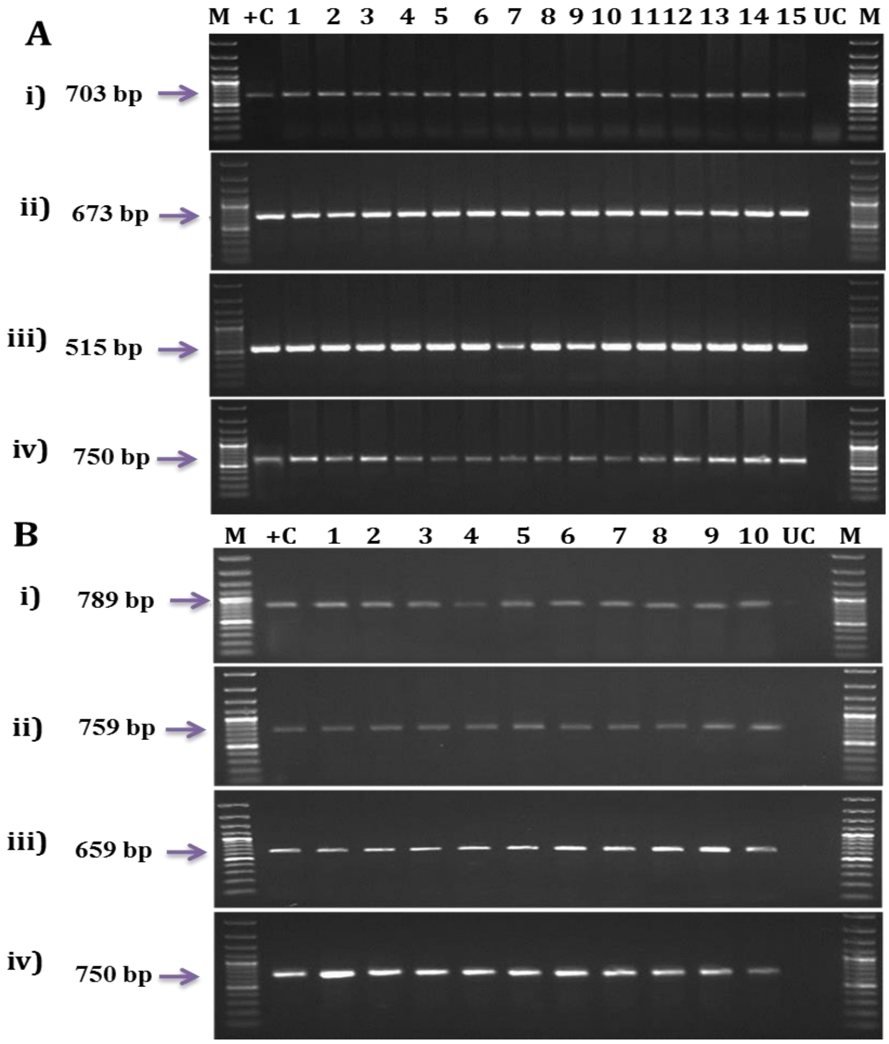

Figure S7. Analysis of T-DNA integration sites in the genome of T_2 eggplant events 18.4 (A) and 20.7 (B) expressing HIGS constructs of *msp-18* and *msp-20*, respectively. Sequenced PCR products depict the location of 35S promoter, right border, adapter and event-specific priming site (bold and underlined) in different colours. Using event-specific primers a 446 bp and a 762 bp fragment was PCR amplified in events 18.4 and 20.7, as well as in their 8 (P1 to P8) and 6 (P1 to P6) progeny plants, respectively. Conversely, other *msp-18*-specific (18.3, 18.5, 18.6, 18.7 and 18.8) and *msp-20*-specific (20.3, 20.6, 20.7, 20.8, 20.9 and 20.10) events failed to detect those fragments. PC – recombinant clones as positive control. WT – wild-type plants. M – 100 bp marker.

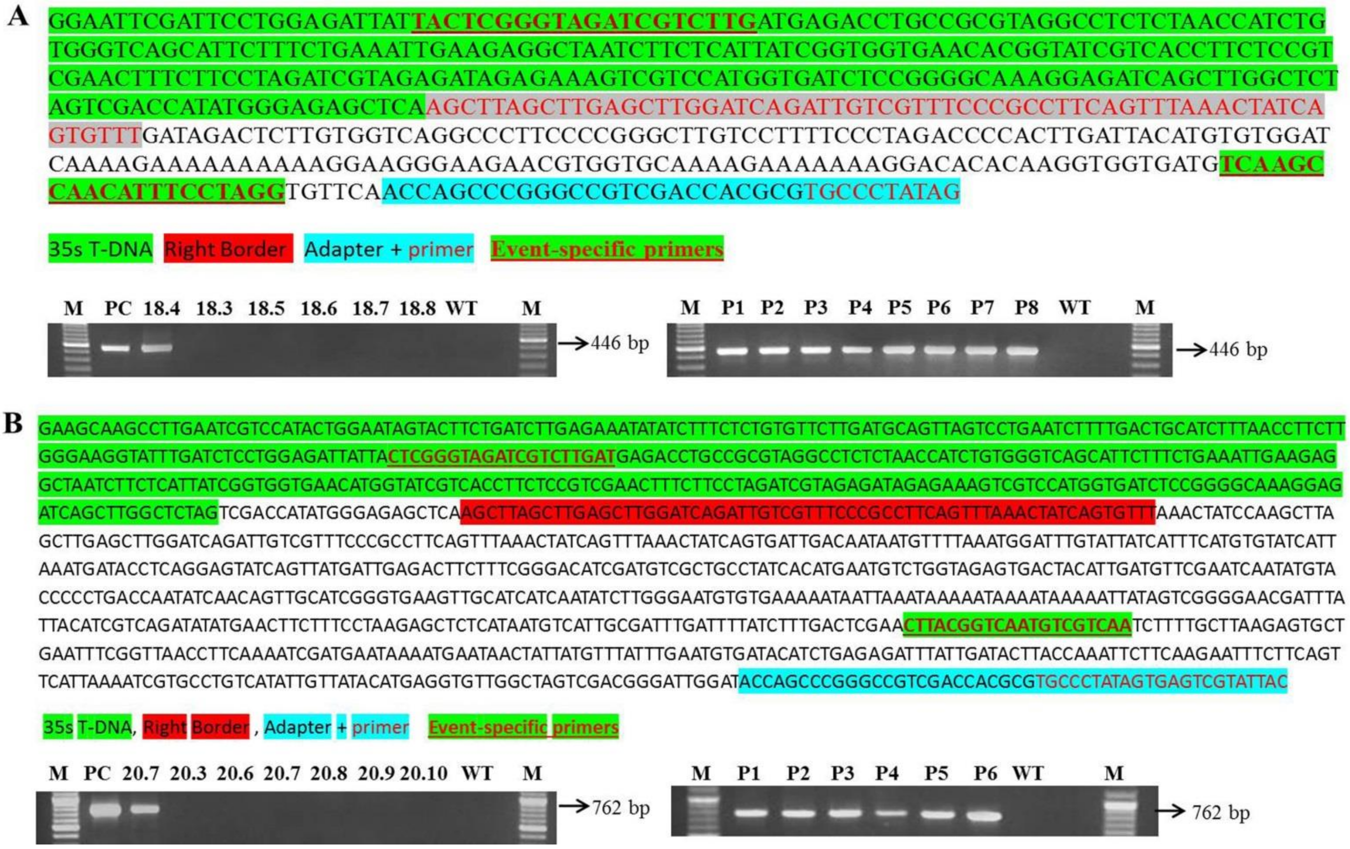

Figure S8. Bioefficacy analysis of T_1 eggplants against *M. incognita*. (A) Intensity of galling in the roots of wild-type (WT), *msp-18*-expressing (18.3, 18.5, 18.6, 18.7, 18.4 and 18.8) and *msp-20*-expressing (20.1, 20.5, 20.11, 20.3, 20.4, 20.6, 20.7, 20.8, 20.9 and 20.10) events at 30 dpi. Scale bar = 5 cm. (B) Comparison of fresh root weight in the infected WT and T_1 plants. T_1 plants have shown greater root mass than the WT plants. Number of triangle (Δ) indicates the number of gene copies in each event.

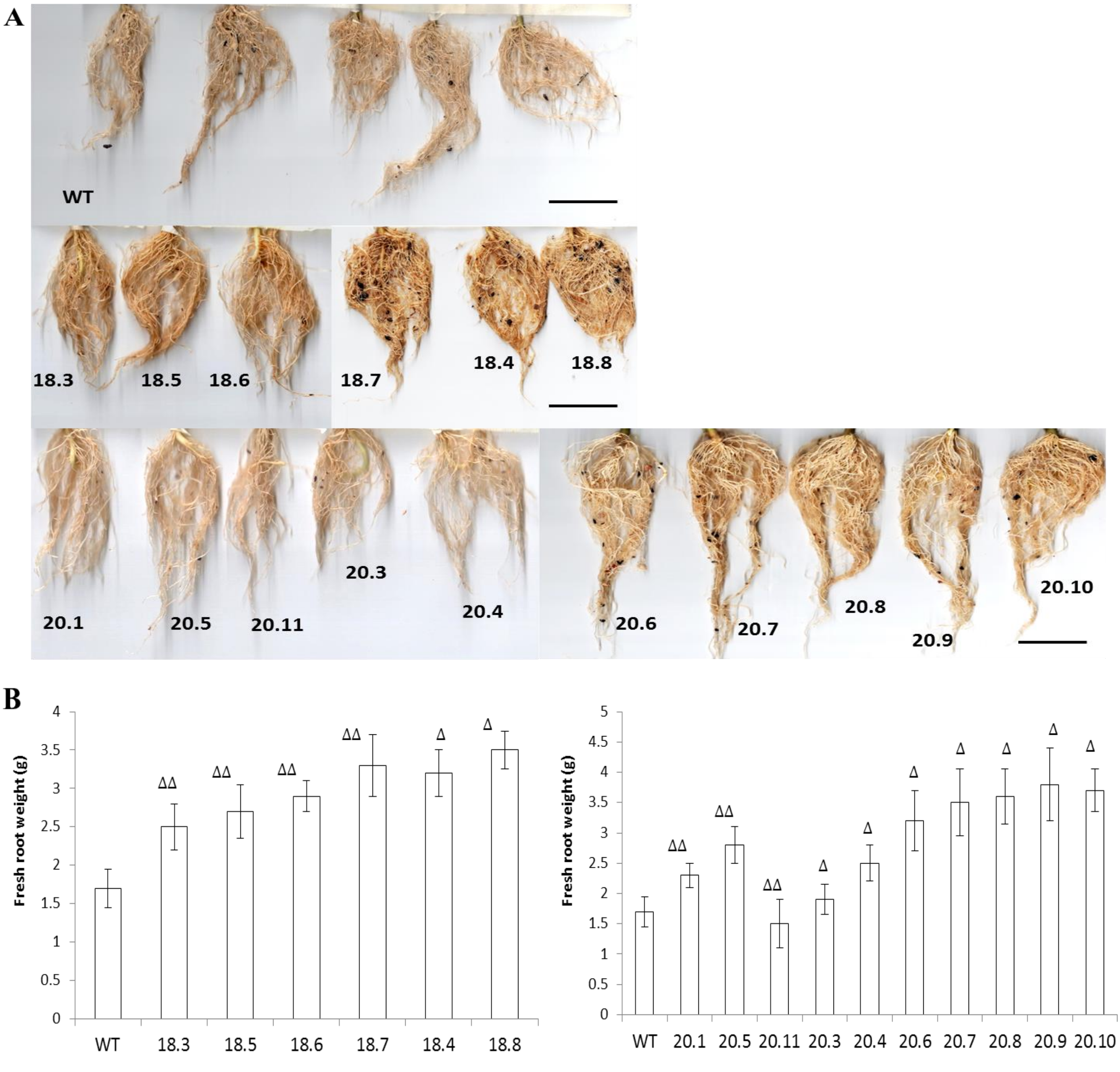

Figure S9. Effect of HIGS of (A) *msp-18* and (B) *msp-20* genes on development and reproduction of *M. incognita* in eggplant. Absolute numbers of galls, egg masses, eggs/egg mass and the corresponding multiplication factor (MF) of *M. incognita* in different T_2 events (*msp-18*-specific: 18.4, 18.8, 18.3, 18.5, 18.6 and 18.7; *msp-20*-specific: 20.3, 20.4, 20.6, 20.7, 20.8, 20.9, 20.10, 20.1, 20.5 and 20.11) and wild-type (WT) plants at 30 dpi. Each bar represents the mean ± SE of n = 6, and bars with different letters (within each parameter) denote a significant difference at P < 0.05, Tukey's test. Number of triangle (Δ) indicates the number of gene copies in each event.

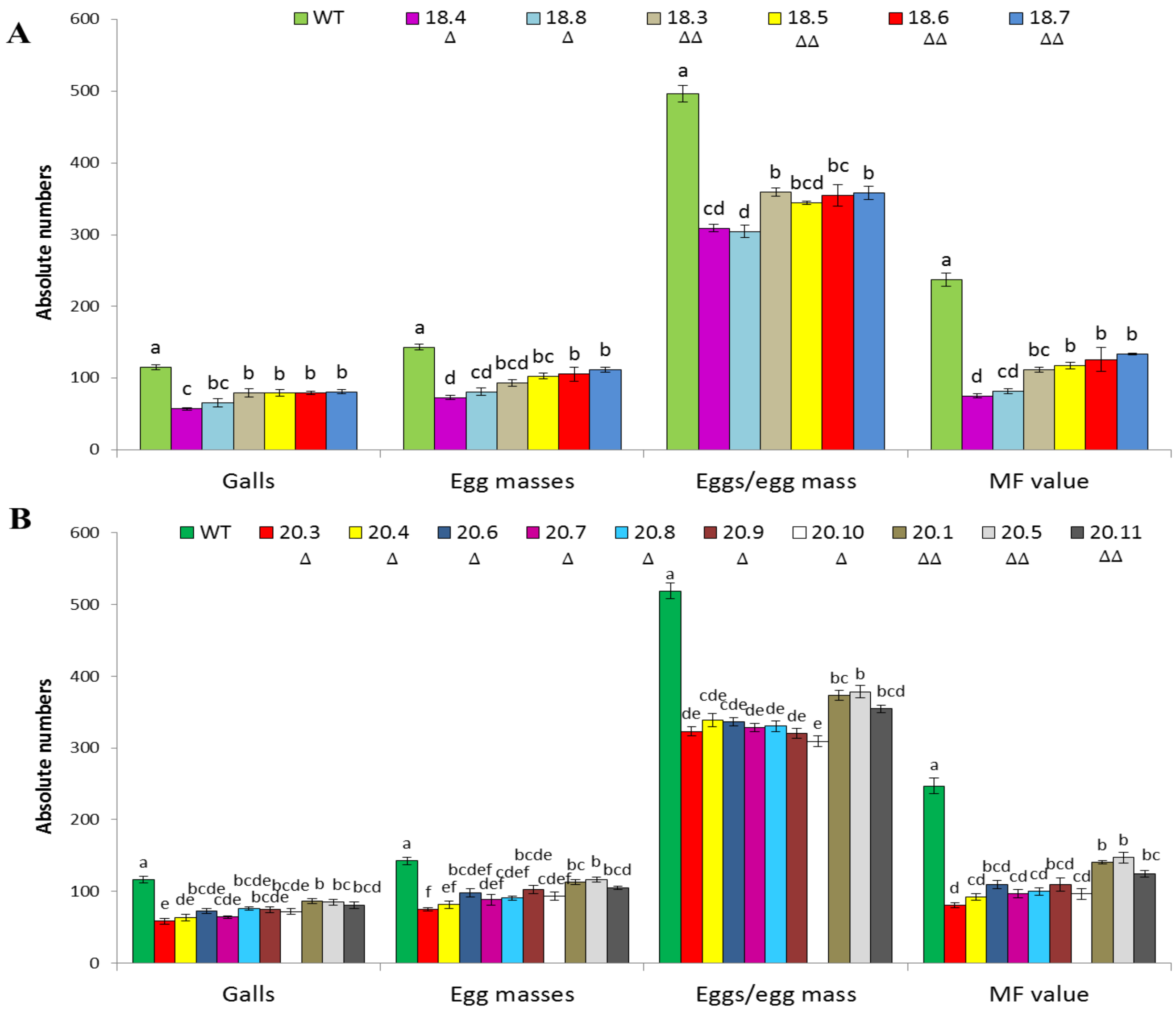

Figure S10. Calculation sheet of ^{14}C incorporation in shoot, root and infecting J2s in a representative sample. Dpm – disintegrations per minute.

Dry weight of test material:
Shoot – 29.5 mg
Root – 15 mg
Infecting J2 (approximately 100 in absolute number) – 0.02 mg

Counts recorded in Liquid Scintillator:
Blank (scintillation cocktail) – 5 dpm
^{14}C labelled shoot – 442,349 dpm
Unlabelled shoot – 12 dpm
^{14}C labelled root – 89,946 dpm
Unlabelled root – 10 dpm
Infecting J2 isolated from labelled plant – 35 dpm
Freshly hatched J2 – 5 dpm

Scintillation counts per mg dry weight in different test material:
Shoot: (labelled-(unlabelled-blank))/29.5 = (442,349-(12-5))/29.5 = 14994.64 dpm
Root: (labelled-(unlabelled-blank))/15 = (89,946-(10-5))/15 = 5996.06 dpm
Infecting J2: (treated-(freshly hatched-blank))/0.02 = (35-(5-5))/0.02 = 1750 dpm

Relative percentage of ^{14}C incorporated in different test material:
Shoot: (14994.64×100)/(14994.64+5996.06+1750) = 65.94%
Root: (5996.06×100)/(14994.64+5996.06+1750) = 26.37%
Infecting J2: (1750×100)/(14994.64+5996.06+1750) = 7.69%

Table S1. List of primers used in the current study.

Gene	Primer name	Primer sequence (5´-3´)	Product Length (bp)	Tm (°C)
**Cloning of *msp-18* and *msp-20* in RNAi vector, genotyping of transgenic plants**				
msp-18 attB	msp-18_attB F	*(AttB1)-TCTTCTCCACCCTCTTCTG	515	60
	msp-18_attB R	*(AttB2)-TCCTTCCCTCCTCCTCACTT		
msp-20 attB	msp-20_attB F	*(AttB1)-TGCCGACTCTGTTGATGTTC	656	60
	msp-20_attB R	*(AttB2)-ACAAGGAGGTGTTGGTGCTT		
CaMV35S promoter	P35 F	TCCTTCGCAAGACCCTTC		
CamV35S terminator	T35 F	CCTTATCTGGGAACTACTCACAC		
nptII	nptII F	CAATCGGCTGCTCTCATGCCG	750	60
	nptII R	AGGCGATAGAAGGCGATGCGC		
**Probes for Southern and northern hybridization assay**				
msp-18	msp-18 F	TCTTCTCCACCCTCTTCTG	456	60
	msp-18 R	TCCTTCCCTCCTCCTCACTT		
msp-20	msp-20 F	TGCCGACTCTGTTGATGTTC	598	60
	msp-20 R	ACAAGGAGGTGTTGGTGCTT		
**Genome walking and event characterization**				
T-DNA specific walker	T-DNA F1	GAAGCAAGCCTTGAATCGTCCATACT G		
	T-DNA F2	CCTGGAGATTATTACTCGGGTAGATC G		
msp-18 T-DNA forward	msp-18 F	TACTCGGGTAGATCGTCTTG	446	60
msp-18 flank reverse	msp-18 R	CCTAGGAAATGTTGGCTTGA		
msp-20 T-DNA forward	msp-20 F	CTCGGGTAGATCGTCTTGAT	762	60
msp-20 flank reverse	msp-20 R	TTGACGACATTGACCGTAAG		

*(attB1): GGGGACAAGTTTGTACAAAAAAGCAGGCT
*(attB2): GGGGACCACTTTGTACAAGAAAGCTGGGT

Table S2. Percentage reduction in different parameters of *M. incognita* development and reproduction on eggplant events (n=6) expressing HIGS constructs of *msp-18* and *msp-20* genes compared to the wild-type plants.

Event number and stage		Number of galls	Number of egg masses	Number of eggs/egg mass	MF value
msp-18-specific events					
18.4	T1	47.29	49.25	40.20	**69.68**
	T2	50.43	49.06	37.71	**68.28**
18.8	T1	44.03	45.03	39.99	**67.04**
	T2	43.23	43.48	38.72	**65.49**
18.3	T1	33.24	39.32	34.79	**60.52**
	T2	30.83	34.88	27.51	**52.86**
18.5	T1	32.43	37.20	34.86	**59.13**
	T2	31.69	28.37	30.67	**50.37**
18.6	T1	32.70	32.98	30.74	**53.66**
	T2	31.41	26.28	28.52	**46.90**
18.7	T1	30.81	27.27	29.66	**48.93**
	T2	29.96	21.85	27.78	**43.64**
msp-20-specific events					
20.3	T1	46.77	45.86	38.48	**66.69**
	T2	49.71	47.42	37.72	**67.30**
20.4	T1	43.01	40.65	32.14	**59.82**
	T2	45.12	42.75	34.63	**62.63**
20.6	T1	36.02	35.21	32.67	**56.41**
	T2	37.64	31.30	35.09	**55.53**
20.7	T1	44.89	40.65	37.82	**63.19**
	T2	44.82	38.08	36.69	**60.82**
20.8	T1	35.48	38.26	36.37	**60.67**
	T2	34.19	36.45	36.31	**59.54**
20.9	T1	37.37	27.60	33.79	**51.97**
	T2	35.92	28.27	38.23	**55.73**
20.10	T1	38.70	35.65	36.50	**59.07**
	T2	37.93	34.57	40.35	**61.01**
20.1	T1	28.22	24.78	26.66	**44.91**
	T2	25.57	20.79	28.02	**43.16**
20.5	T1	30.11	22.17	25.21	**41.74**
	T2	26.72	18.22	27.05	**40.40**
20.11	T1	28.50	28.48	27.85	**48.39**
	T2	30.17	26.16	31.68	**49.64**
